# Slow data public health

**DOI:** 10.1007/s10654-023-01049-6

**Published:** 2023-10-03

**Authors:** Arnaud Chiolero, Stefano Tancredi, John P. A. Ioannidis

**Affiliations:** 1 Population Health Laboratory (#PopHealthLab), University of Fribourg, Route Des Arsenaux 41, 1700 Fribourg, Switzerland; 2 School of Population and Global Health, McGill University, Montreal, Canada; 3 Institute of Primary Health Care (BIHAM), University of Bern, Bern, Switzerland; 4 Departments of Medicine, of Epidemiology and Population Health, of Biomedical Data Science, and of Statistics, Meta-Research Innovation Center at Stanford (METRICS), Stanford University, Stanford, USA

**Keywords:** Surveillance, Big data, Infodemic, Evidence-based public health

## Abstract

Surveillance and research data, despite their massive production, often fail to inform evidence-based and rigorous data-driven health decision-making. In the age of infodemic, as revealed by the COVID-19 pandemic, providing useful information for decision-making requires more than getting more data. Data of dubious quality and reliability waste resources and create data-genic public health damages. We call therefore for a slow data public health, which means focusing, first, on the identification of specific information needs and, second, on the dissemination of information in a way that informs decision-making, rather than devoting massive resources to data collection and analysis. A slow data public health prioritizes better data, ideally population-based, over more data and aims to be timely rather than deceptively fast. Applied by independent institutions with expertise in epidemiology and surveillance methods, it allows a thoughtful and timely public health response, based on high-quality data fostering trustworthiness.

## Introduction

Are we drowning in the massive volume of information coming from surveillance and public health-related research? This may sound counterintuitive because common wisdom in public health assumes that we need more data [1–3]. It is also at odds with the idea that data sciences and big data, notably through the developments of digital health, artificial intelligence, personalized medicine, and precision public health, will be the decisive transformative element of public health [3–5]. In this essay, we argue, however, that getting more data will not be sufficient to solve our information needs for public health decision-making. As revealed by COVID-19 pandemic surveillance failures, we need better data and well-defined information needs, within a population perspective. Data of dubious quality and reliability waste resources and create new data-genic public health damages [6]. We call, therefore, for a slow data public health to foster evidence-based and high-quality data-driven public health decision-making.

### Failure of surveillance systems

The COVID-19 pandemic was a crash test, revealing the state of surveillance systems across the world and how research informs public health decisions [7]. In the early phase of the pandemic, many countries' surveillance and health information systems were not ready for such a threat [7]. Systems to capture timely and high-quality data useful for decisions were missing, and the lack of common standards hampered international coordination and comparisons. With time, the situation improved with the growing availability of multiple types of data and the development of more efficient surveillance systems. Nevertheless, data ingestion workflow remained problematic, and major inconsistencies across countries and settings continued, including, e.g., for death outcomes with intractable between countries differences in definitions, completeness, and over- or under-counting [8, 9].

Furthermore, on top of the pandemic, an epidemic of information, that is, an “infodemic”, took place [10]. Surveillance-related numbers flooded society through multiple media. Probably at an unprecedented scale, numerous researchers started to work on COVID-19 surveillance-related topics. The volume of surveillance and research data became rapidly overwhelming, also due to their massive echo through media and digital social platforms accompanied by often gross distortion from conflicted stakeholders and conspiracy theories. The convergence of high-volume and low-quality data became a major problem for policymakers and the public, with information needs only partly fulfilled.

### Confusion between surveillance and research

Behind the failure of surveillance and information systems are three major problems that must be fixed if we want to move toward evidence-based and rigorous data-driven public health decision-making (Table 1). The first problem is the confusion between surveillance and research [1, 11]. Many research findings have been used as information for decision-making. Research provides knowledge and helps bound uncertainties around this knowledge, and that can be useful to guide decisions. However, it is not designed explicitly to inform policy-making and support public health decisions. It is the role of surveillance to help decision-making through the systematic collection, analysis, and interpretation of data, integrated with the timely dissemination of the resulting information to those responsible for preventing and controlling disease [1].

**Table 1:** Three problems magnified by the pandemic and hampering the application of evidence-based and rigorous data-driven health decision-making

Problem	Characteristics	Solution
1. Confusion between surveillance and research	Poor knowledge by researchers of surveillance activities and of policymakers’ needs	Increase surveillance culture among researchers
	Weak health data literacy of decision-makers	Foster collaboration between policymakers, surveillance experts, and researchers
		Rely for surveillance on independent and scientific institutions with expertise in epidemiology and surveillance methods
2. Big data do not speak by themselves	Poor quality of (organic) data	Train public health experts in measurement issues (methods, type of error, performance)
	Difficulty to characterize source population (selectivity bias)	Evaluate and document data quality systematically
	Diagnosis-based rather than population-based data	
		Characterize the study and target population, the sampling method, and the representativeness
		Improve data integrity, completeness, consistency, and quality
		Build surveillance systems to catch population-level data
3. Infodemic	High volume of data and information	Improve research quality and evidence synthesis production
	Multiple data sources and information producers	
		Track and debunk misinformation
	Misinformation spreading	Train policymakers in surveillance and health data science
	Doubts on the reliability of information and of experts, as well as on the independence of institutions producing information	
		Identify reliable experts and scientific institutions working in an evidence-based framework, not exposed to political influences

With the pandemic, many researchers and data scientists were for the first time involved in surveillance-related research, counseling, and policymaking, while experiencing intense media exposure. On the one hand, this was useful in some settings, resulting in greater democratization of surveillance (done not only through governmental agencies), supporting diverse analytic approaches, and external testing. On the other hand, much noise—if not misinformation—emerged from this exposure, notably because researchers are not trained for public health counseling and communication activities; they often lack a public health surveillance culture, and they tend to be overconfident about how they understand others (citizen, decision-makers) and how others understand them [12]. Concurrently, many decision-makers, while struggling with their weak health and data literacy, had to deal for the first time with researchers and the convoluted scientific processes of knowledge production. The pandemic made visible this process in real-time and the presence of scientific discourse in the public sphere was stronger than ever [1].

### Fooled by big data

The second major problem revealed by the pandemic is that big data do not speak by themselves. Big data refer to the massive amount of data accessible through the digitalization of all aspects of life, including health and healthcare [4]. They are characterized by their variety, volume, and velocity—the 3 Vs—but also often by their poor (or undocumented) quality [1]. To better understand the issue of the poor quality of these data, it is helpful to make a distinction between “designed” and “organic” data [13].

Surveillance activities are traditionally based on what is called “designed” data coming from classical surveillance tools such as surveys or registries and ideally gathered using well-defined epidemiologic methods to capture population-level data. The validity and reliability of these data can be documented, and they can be tailored to address specific public health problems. Conversely, a large share of big data can be characterized as “organic” because they are a byproduct of another activity, e.g., health care provision [13]. While their secondary use makes them potentially informative for surveillance, their validity and reliability are often only partially documented. A growing share of research is also conducted with organic data, opening new avenues, notably in the field of health services research and “precision” public health. Data-driven analyses (such as data mining or applications of artificial intelligence) make these data fit to produce information useful for decision-making. However, some features of these methods (e.g., flexible data analysis, multiplicity of options, and lack of prespecified hypotheses) increase the probability of false findings [14].

Beyond their intrinsic value to capture any information of interest, organic data are typically exposed to strong “selectivity bias”, a term coined to highlight that the source population of these data is difficult to identify and is not stable across time and settings (Table 2) [1]. The population perspective is blurred. Completeness and representativeness cannot be ensured due to the non-probabilistic nature of these data and the selectivity of people from which data are recorded [15]. Because the source population and its sampling circumstances change, these data are constantly evolving, making them problematic for surveillance. Information derived from these data is not easily transportable to a target population (Box 1).

**Table 2:** Selected practices to improve the quality of research related to surveillance (adapted from Ioannidis 2014 [14]) and their relevance for a slow data public health

Research practices	Relevance for a slow data public health
Large-scale collaborative research	Research and surveillance benefit from coordination of efforts and collaboration in the identification of needs with standardization in data collection methods across different sources
	Critical for comparisons and benchmarking
Adoption of replication culture	To enhance reproducibility, especially under conditions of massive research outputs
	In a quality improvement framework, to provide feedback to surveillance systems for their continuous improvement
Containment of conflicted sponsors and authors	To foster trust in surveillance expertise and evidence
	To protect surveillance activities from political influence
	To avoid academic militantism blurring the boundary between politics and science
More appropriate statistical methods, and standardization of definitions and analyses	Highly relevant as data become more complex and error-prone and as many information producers are involved
	For surveillance, favor methods that are clear enough for dissemination to allow informed decision-making
	Give more weight to metrology training [18]
More stringent thresholds for claiming discoveries or ‘‘successes’’	Essential for efficient dissemination of information and to prevent wasting resources on trivial or biased information
	To prevent exaggerated information, excessive excitement, and eventual disappointment at the time of dissemination
	To enhance trust with proper and honest communication of uncertainty
Improvements in peer review, reporting, and dissemination of research	For surveillance, the processes of reporting and dissemination have to be explicitly defined a priori
	Mediatization of surveillance and study results can create sensationalism and should be done cautiously – to avoid “medicine by press release”
	Requires independent and scientifically credible institutions with experts trained in epidemiology and surveillance methods [24]

Another issue with big data is that their size gives a false sense of security [19]. Indeed, increasing data size shrinks confidence intervals around estimates but solidifies the effect of survey bias. This has been called the “Big Data Paradox”: the more data we have, the more we can be fooled by “precisely inaccurate” estimates [20]. Hence, surveys on Facebook, with about 250′000 responders per week, could estimate first-dose COVID-19 vaccine uptake in US adults with minuscule margins of errors but in excess of 17 percentage points compared to CDC estimates [19].

### Infodemic management

The third major problem magnified by the pandemic is the “infodemic”, a term coined to describe the overabundance of information, including misinformation, disseminated on a large scale via multiple (mostly non-scientific) channels [21]. The spreading of data and information is accelerated through direct communication and content production by social media platforms, without the mediation of relevant experts [10]. In addition to the growing sources of data, there is a multiplication of information producers, a phenomenon favored by emerging artificial intelligence tools [6]. Moreover, the echo chamber mechanism reinforces shared narratives and fosters individual polarization [10]. Decision-making under these circumstances becomes challenging, as policymakers and citizens try to navigate the mounting pressure from the infodemic that affects public opinion, perceptions, and expectations. The pandemic has therefore highlighted how critical it is to identify reliable experts and to define what can reasonably be expected from them (Box 2).

**Box 1:** Data from populations or populations from data?

Usually in epidemiology and public health, data are sampled from a well-defined study population. In the case of simple random sampling, and if there are no major internal validity issues, estimates from the data can be easily generalized to the study population [16]. In an age of big data and digital health, data are rarely sampled randomly from a well-defined population. In some cases, the data come from the whole population, and it offers some advantages for surveillance [17]. In other cases, and that is problematic, the population generating the data is elusive and changing like a moving target, and it is not possible to identify clearly what sampling mechanisms produce the data. In a growing number of settings, data are used per se without referring explicitly to a well-defined population. For instance, while social media data are increasingly used for surveillance and public health research, they do not provide information on a well-defined target population beyond the users of social media at the time of the study. The concepts of representativeness, generalizability, and transportability become fuzzy. If there is no transportability to a target population, the data are less useful for decision-makers aiming to improve the health of this population. At the extreme, when data are the population, the risk is to make decisions based on dataflows cut off from shared values and genuine information needs, which leads to “dataism” [18]	

In response to huge volume of research output and its reverberation through multiple venues, evidence syntheses are needed to offer a balance against the untamed infodemic. Hence, multiple systematic or rapid reviews have been produced to summarize COVID-19-related research, but too many were of low quality [25]. Further, many COVID-19 study findings were the subject of exaggerated information and major excitements followed by severe disappointments, as well as by rapidly alternative extreme and opposite research claims [26]. For example, interventions that eventually were shown to be ineffective or harmful had some early studies claiming extremely promising results and receiving high media attention; they had also high citations in the scientific literature, and a prominent place in reviews and scientific opinion pieces [26]. All that noise blurred evidence-based decision-making.

One consequence of infodemic is that the efficiency of public health information systems, as defined by the ratio of useful information over resources allocated to gather it, diminishes enormously. Low-cost data end up causing huge costs by misguiding both the surveillance and research enterprises as well as decision-making (Box 3).

**Box 2:** Who are the experts? And where are they?

The expectations from experts were huge and not reasonable during the COVID-19 crisis. Rapid and valid responses to all kinds of pressing questions were expected from citizens and health authorities. However, especially at the start of the pandemic, the knowledge was insufficient to have responses to many of these questions, but experts were pressured to give their opinion, taken too often as grounded on solid evidence while it was built from limited observations and weak hypotheses – if not from common sense and gut feelings. Different scientific disciplines (e.g., epidemiologists versus virologists) competed for interpretive authority regarding the COVID-19 pandemic. Surprisingly, all subfields of science had scientists who published on COVID-19, often venturing far from their field of expertise (e.g., physicists or mechanical engineers) [22]. Many scientists who published on COVID-19 epidemiology had no training in epidemiology and public health surveillance methods. For most of the “experts” who appeared prominently in the media, there was a worrisome disconnect between claimed media expertise and actual population health science expertise [23]. Identifying relevant experts who could be trusted was a major problem, notably due to the growing distrust of scientific institutions in charge of public health surveillance activity. This calls to strengthen the autonomy and credibility of scientific institutions producing surveillance evidence, first, through adequate staff training in epidemiology and surveillance methods and, second, by maintaining a separation between these institutions and governments using this evidence to design policy. One must learn from the failure of the CDC on COVID-19, in part due to political interferences [24]. Highly credible scientific institutions are also necessary for experts from different domains to work together, despite different and evolving views on the evidence	

The waste of resources, debates, and the recycling of wasteful information can slow the identification and implementation of effective evidence-based policies. Further, notably through the spread of misinformation and fake news eroding trust in institutions, communities may eventually reject sound expert advice and evidence-based policymaking, as they become difficult to differentiate from the surrounding waste. New developments in artificial intelligence could accentuate these trends as they can act as multipliers of the infodemic [6].

### Toward a slow data public health

**Fig. 1:**
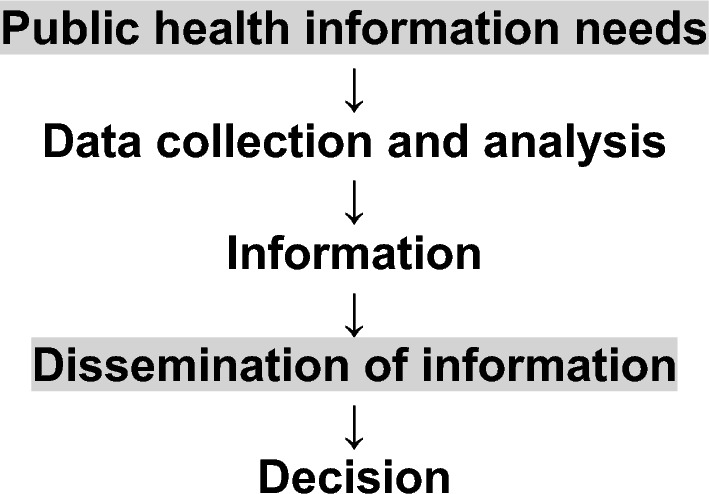
Within a surveillance framework [1], the process of public health information production goes from the identification of information needs, at a population level, to decision-making. In a slow data public health approach, it is critical to be clear and purposeful regarding the information needs and the dissemination strategy

In response to these challenges, we propose to move from big data public health to slow data public health [6]. The pandemic has revealed how a massive amount of research and surveillance data is not sufficient to fulfill our information needs. Slow data means that what matters more than data collection and analyses are, first, the careful and purpose-driven identification of specific public health information needs and, second, the efficient and purpose-driven dissemination of this information (Fig. 1). It also highlights the importance of collecting fewer, but higher quality data designed for these purposes.

Identifying public health information needs requires surveillance experts, data scientists, healthcare providers, patient representatives, researchers, policymakers, and citizens to work together. Policymakers and citizens are the legitimate stakeholders to define the needs and which health outcomes should be valued. They also determine which resources are given to surveillance and research activity [6]. Surveillance experts, working in independent and scientifically credible institutions [24], are key players in linking policymakers and health data researchers. Once the needs are identified, these experts can design adequate surveillance systems. Anticipation is also a key feature of slow data public health, and potential information needs must be defined early on, not only during a crisis [29].

A purpose-driven information dissemination strategy is the other central element of a slow data public health. Transforming evidence into useful information for decision-making requires putting communication, dissemination, and implementation sciences at the heart of surveillance [30]. The infodemic has also revealed that the cognitive satisfaction provided by the information, more than its quality, favors its diffusion [31]. People understand people and stories more than data; they need narratives around the data. We have therefore to develop a shared culture of public health surveillance between researchers, data scientists, and decision-makers. In an era of infodemic and misinformation, and because the truth does not defend by itself in a deregulated information and cognitive market [31], maintaining trust in public health expertise and in science is essential in this effort. Scientists and decision-makers must be better trained in surveillance and epidemiology. At a societal level, it will also necessitate improving health data literacy and critical thinking.

To improve the quality of surveillance-related research, we know the measures we should apply (Table 2), and several of these measures are integral to a slow data approach. Efforts need to be coordinated at national and international levels, with standardization of definitions and practices to strengthen the comparability of the data eventually collected across different places and over time. Institutions expert in surveillance and population health sciences should lead initiatives for the integration of surveillance data streams and designing foundational data for surveillance. The impact of poor standardization should be fully acknowledged, and more weight should be given to metrology [18]. A reproducibility culture is also very helpful, especially to prevent the dissemination of exciting claims, rapidly gaining numerous believers, as it happened for many proposed treatments for COVID-19 [26]. The whole effort should also give priority to trust, and uncertainty should be communicated to its full breadth. Overpromising certainty to justify public health measures is likely to backfire. Finally, the mediatization of surveillance and study results can create sensationalism and should be done cautiously.

**Box 3:** Surveillance bias

Diagnoses data from healthcare providers are increasingly used for surveillance [27]. Diagnoses trends can differ from diseases trends, and ignoring this difference can lead to a surveillance bias [1]. Hence, during the pandemic, the number of reported COVID-19 cases has been routinely used as the main indicator of the infection's spread severity, because this data was readily available daily and, at least apparently, relatively simple to communicate. However, the difference between the diagnosis (based on a positive test) and the disease we want to prevent (severe symptomatic infection) was often overlooked. Focusing on the number of positive tests to gauge the severity of the pandemic could be misleading since this number was influenced over time by changes in test availability and testing intensity, as well as changes in reporting rates. As a result, the changes in the number of cases diagnosed did not match in a predictable way with the number of clinical diseases. Another example is the use of hospitalizations as a marker of epidemic severity, which is hampered by the fact that the threshold for hospitalization changed due to changing perceptions of risk, attitudes about who should be hospitalized, beliefs about the efficacy of in-hospital treatments, and different incentives to hospitalize patients with SARS-CoV-2 infection. Comparing naively the hospitalization rate over time or across settings can bias the alleged severity of the pandemic. Prevention of surveillance bias requires strengthening standardization in the definition of health events as well as building information systems to capture high-quality population-based data, and not only diagnosis-based data. When multiple sources of data are available, each with its own bias, triangulation can also help [28]. It can, however, further compound bias especially when there are dominant (but false) narratives; then, new biases are added to the argumentation to continue supporting false premises	

To strengthen surveillance outputs, the information production machinery should be conceptualized within the ecosystem of health decision-making [32], in a population perspective. Within a metrology framework [18], fostering primary data providers to improve the structure and semantics of the data they collect is critical to produce meaningful information from them [6]. Further, while data from healthcare providers constitute the basic layer of surveillance, the core surveillance activity should be designed at a population level, using population-based tools with information systems designed to avoid surveillance bias. Finally, slowing information production does not imply poor timeliness, because once we know which information is needed and how to disseminate it, the production process is more efficient.

## Conclusion

In the age of infodemic, providing useful information for decision-making requires more than getting more data. Data of dubious quality and reliability waste resources and create data-genic public health damages. A slow data public health means, first, prioritizing the identification of specific information needs and, second, disseminating information in a way that informs decision-making, rather than devoting massive resources to data collection and analysis. It requires better data, ideally population-based, rather than more data, and aims to be timely rather than deceptively fast. Applied by independent institutions with expertise in epidemiology and surveillance methods, it allows a thoughtful and timely response, based on high-quality data fostering trustworthiness.
